# 2‐deoxyglucose and β‐hydroxybutyrate fail to attenuate seizures in the betamethasone‐NMDA model of infantile spasms

**DOI:** 10.1002/epi4.12561

**Published:** 2021-11-24

**Authors:** Remi Janicot, Li‐Rong Shao, Carl E. Stafstrom

**Affiliations:** ^1^ Division of Pediatric Neurology The Johns Hopkins University School of Medicine Baltimore Maryland USA

**Keywords:** epileptic encephalopathy, glycolysis, ketogenic diet, ketone body, metabolism

## Abstract

Infantile spasms (IS) is an epileptic encephalopathy with a poor neurodevelopmental prognosis, and limited, often ineffective treatment options. The effectiveness of metabolic approaches to seizure control is being increasingly shown in a wide variety of epilepsies. This study investigates the efficacy of the glycolysis inhibitor 2‐deoxyglucose (2‐DG) and the ketone body β‐hydroxybutyrate (BHB) in the betamethasone‐NMDA model of rat IS. Prenatal rats were exposed to betamethasone on gestational day 15 (G15) and NMDA on postnatal day 15 (P15). Video‐electroencephalography (v‐EEG) was used to monitor spasms. NMDA consistently induced hyperflexion spasms associated with interictal sharp‐slow wave EEG activity and ictal flattening of EEG signals, reminiscent of hypsarrhythmia and electrodecrement, respectively. 2‐DG (500 mg/kg, i.p), BHB (200 mg/kg, i.p.), or both were administered immediately after occurrence of the first spasm. No experimental treatment altered significantly the number, severity, or progression of spasms compared with saline treatment. These data suggest that metabolic inhibition of glycolysis or ketogenesis does not reduce infantile spasms in the NMDA model. The study further validates the betamethasone‐NMDA model in terms of its behavioral and electrographic resemblance to human IS and supports its use for preclinical drug screening.

## 1 INTRODUCTION

Infantile spasms (IS) is an epileptic encephalopathy associated with cognitive impairment and poor neurodevelopmental outcome.^1^ It remains unclear how the over 200 etiologies linked to IS converge to the common clinical manifestation of flexion or extension spasms.^2^ IS has unique EEG characteristics, with interictal high amplitude, chaotic, slow‐ and sharp‐wave activity (hypsarrhythmia), and a sudden, generalized reduction in voltage (electrodecrement) during an actual spasm.^3^ IS does not respond to most conventional antiseizure medications but may respond to adrenocorticotrophic hormone, corticosteroids, or vigabatrin. To conclude treatment efficacy, both the spasms and hypsarrhythmia must be eliminated.^4^ Unfortunately, current first‐line treatments are often associated with severe side effects and are only effective in ~70% of cases,^5^ leaving many IS patients with a condition that exacts a huge medical and societal toll.

Metabolic interventions such as the ketogenic diet (KD) are promising therapies in IS, especially in patients resistant to first‐line treatments.^6^ This high‐fat, low‐carbohydrate diet leads to the production of ketone bodies (KB) (acetone, acetoacetate, BHB) as products of fatty acid oxidation. KB may exert broad‐spectrum antiseizure effects by altering neurotransmitter release, ion channel function, or other mechanisms.^7^ The low‐carbohydrate component of the diet plays a critical role in the KD's antiseizure effect. Seizure activity is highly energy‐dependent and thus susceptible to metabolic intervention such as glycolysis inhibition—a small amount of carbohydrate intake quickly reverses the KD's antiseizure effect.^8^ We have shown previously that the glycolysis inhibitor 2‐deoxyglucose (2‐DG) abates seizures in multiple in vitro and in vivo seizure models.^9^, ^10^, ^11^, ^12^

Because of the heterogeneous etiologies and the uniqueness of IS clinical and EEG features, it has been challenging to establish an animal model that recapitulates all human symptoms and EEG findings of IS.^13^, ^14^ Therefore, an animal model that meets certain criteria and mimics some of the clinical features of IS would be informative for understanding the pathophysiology and developing new therapies.^15^ The model developed by Velíšek and colleagues combines prenatal betamethasone administration (a prenatal stressor affecting the hypothalamic‐pituitary axis) and postnatal treatment with the glutamate receptor agonist N‐methyl‐D‐aspartate (NMDA) to elicit spasms; this acute model recapitulates several behavioral, EEG, and therapeutic features of cryptogenic IS: spasms, ictal electrodecrement, and responsiveness to ACTH.^16^ The model's reproducible seizure semiology provides an advantage for drug screening. Using this model, we hypothesized that acute treatment with 2‐DG, BHB, or their combination would reduce the severity or frequency of spasms.

## 2 METHODS

All procedures used in this study were approved by the Institutional Animal Care and Use Committee of Johns Hopkins University.

### 2.1 Prenatal betamethasone treatment and postnatal EEG electrode implantation

Sprague‐Dawley dams (Envigo International Holdings, NJ) received two injections of betamethasone (0.5 mg/kg each, i.p., at 7:30 am and 5:30 pm) on gestational day (G) 15. Dams were kept in the animal facility and usually gave birth to offspring on G21‐22. Litter size averaged 5‐10 pups; they gained weight and grew normally.

**FIGURE 1:**
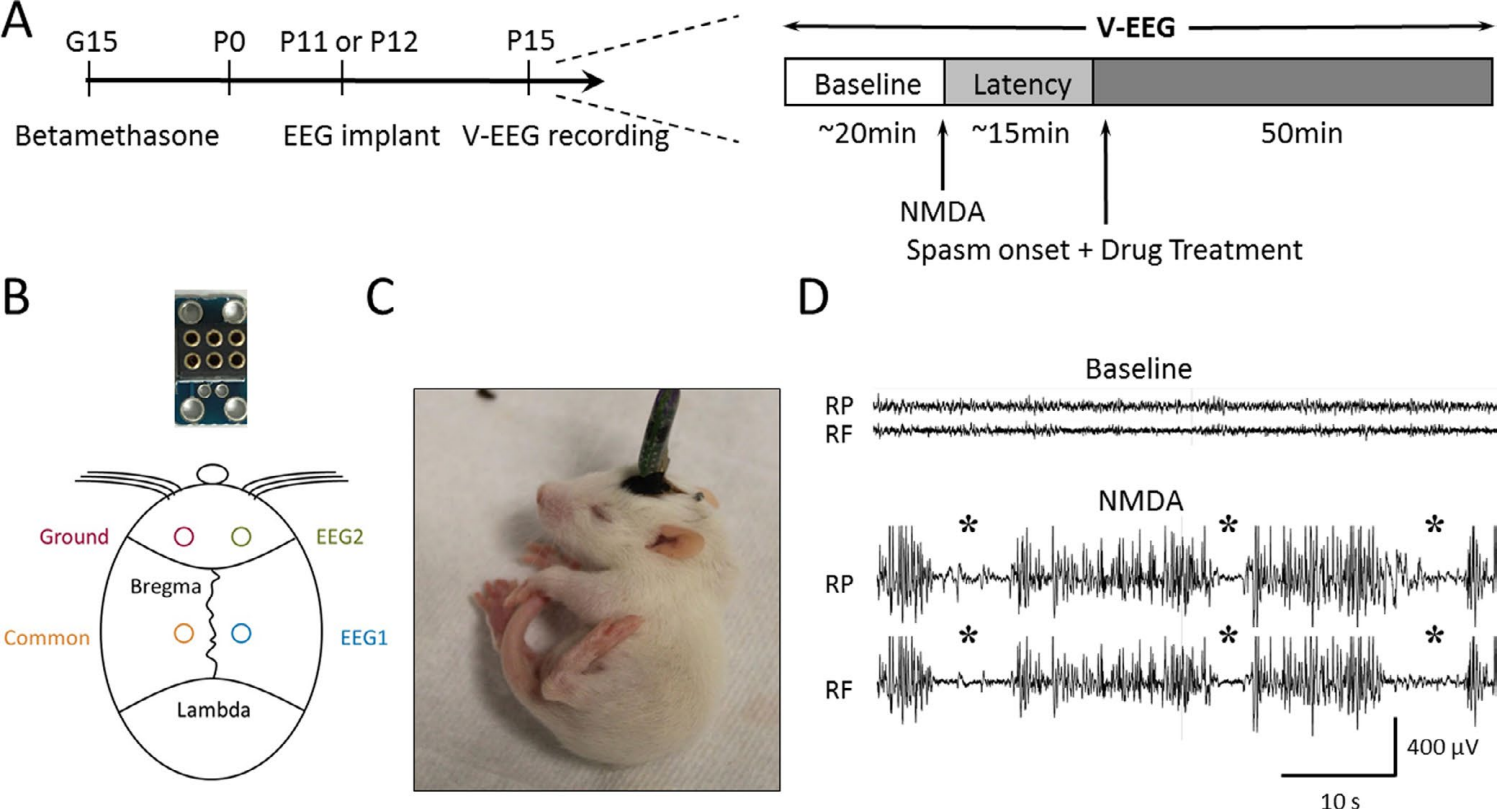
Experimental protocol and NMDA‐induced spasm phenotypes. (A) diagram showing the experimental design and procedure. Dams were exposed to betamethasone at G15. After birth, rat pups were implanted with EEG electrodes on P11‐12 and were given NMDA on P15 and monitored with vEEG. After 20 min of baseline v‐EEG recording, rats were injected with NMDA (15 mg/kg, i.p.) to induce spasms. After a latency period, the first spasm occurred and one of the following treatments was then immediately given: saline (i.p.), 2‐DG (500 mg/kg, i.p.), BHB (200 mg/kg, i.p.), or 2‐DG + BHB (500 mg/kg 2‐DG, 200 mg/kg BHB, i.p.). The recording continued until spasms and EEG abnormalities diminished (~50 min after the onset). (B) upper, photograph showing the miniature headmount used in the study with 4 large holes for electrode implantation and 6 pin holes for connection to preamplifier; bottom, diagram showing the approximate locations of EEG electrodes. (C) photograph showing a typical behavioral phenotype during NMDA‐induced spasm. Rats usually fell on their sides and the tail coiled under the body while the neck and head bent in hyperflexion. Spasms typically lasted ~8 s. (D) representative traces showing NMDA‐induced electrographic phenotype of spasms. The top traces represent the raw EEG baseline recording before NMDA injection in parietal (RP) and frontal (RF) cortices. The bottom traces show the EEG during NMDA‐induced spasms (marked by asterisks). Note the interictal EEG activity was increased and mixed with irregular slow and sharp waves, while the occurrence of spasms was associated with a sudden voltage reduction (ie, electrodecrement)

EEG electrodes were implanted on postnatal day (P)11 or P12. Pups were anesthetized with ketamine‐xylazine (40‐60 mg/kg ketamine; 3‐5 mg/kg xylazine, i.p.) and placed on a heating pad. The skull was cleaned with 3% hydrogen peroxide and a prefabricated miniature EEG headmount (Figure 1B, Pinnacle Technology) was placed along the midline between bregma and lambda. Four stainless‐steel mini‐screws (0.12″) were inserted into 4 burr holes to secure the headmount and serve as recording electrodes (1 ground, 1 EEG common, and 2 EEG). The two electrodes were located over the frontal and parietal cortical areas of the right hemisphere ~1.5 mm lateral to the midline and 6 mm apart (Figure 1B). The headmount was fixed using dental acrylic and the incision was closed using a surgical clip. Pups were left on the heating pad until they recovered from anesthesia, at which time they were returned to their cage with the dam.

### 2.2 Spasms induction, recordings, and treatments

On P15, pups were placed individually in a plexiglass chamber and their headmounts were connected to a tethered v‐EEG recording system (Pinnacle). After 15‐20 min of baseline recording, NMDA (15 mg/kg, i.p.) was administered to induce spasms. Immediately after the first spasm, rats received one of the following treatments (i.p.): (1) saline; (2) 2‐DG (500 mg/kg); (3) BHB (200 mg/kg); and (4) 2‐DG (500 mg/kg) and BHB (200 mg/kg). 2‐DG and BHB doses were chosen based on previous studies.^10^, ^17^ Recordings continued for ~50 min until both spasms and EEG activity diminished. The experimental protocol is illustrated in Figure 1A. EEG signals were acquired using Sirenia Acquisition software (Pinnacle), filtered with a high‐pass filter of 0.5 Hz and a low‐pass filter of 40 Hz, and digitized at 250 Hz. All chemicals were purchased from Sigma.

### 2.3 Analysis and statistics

**FIGURE 2:**
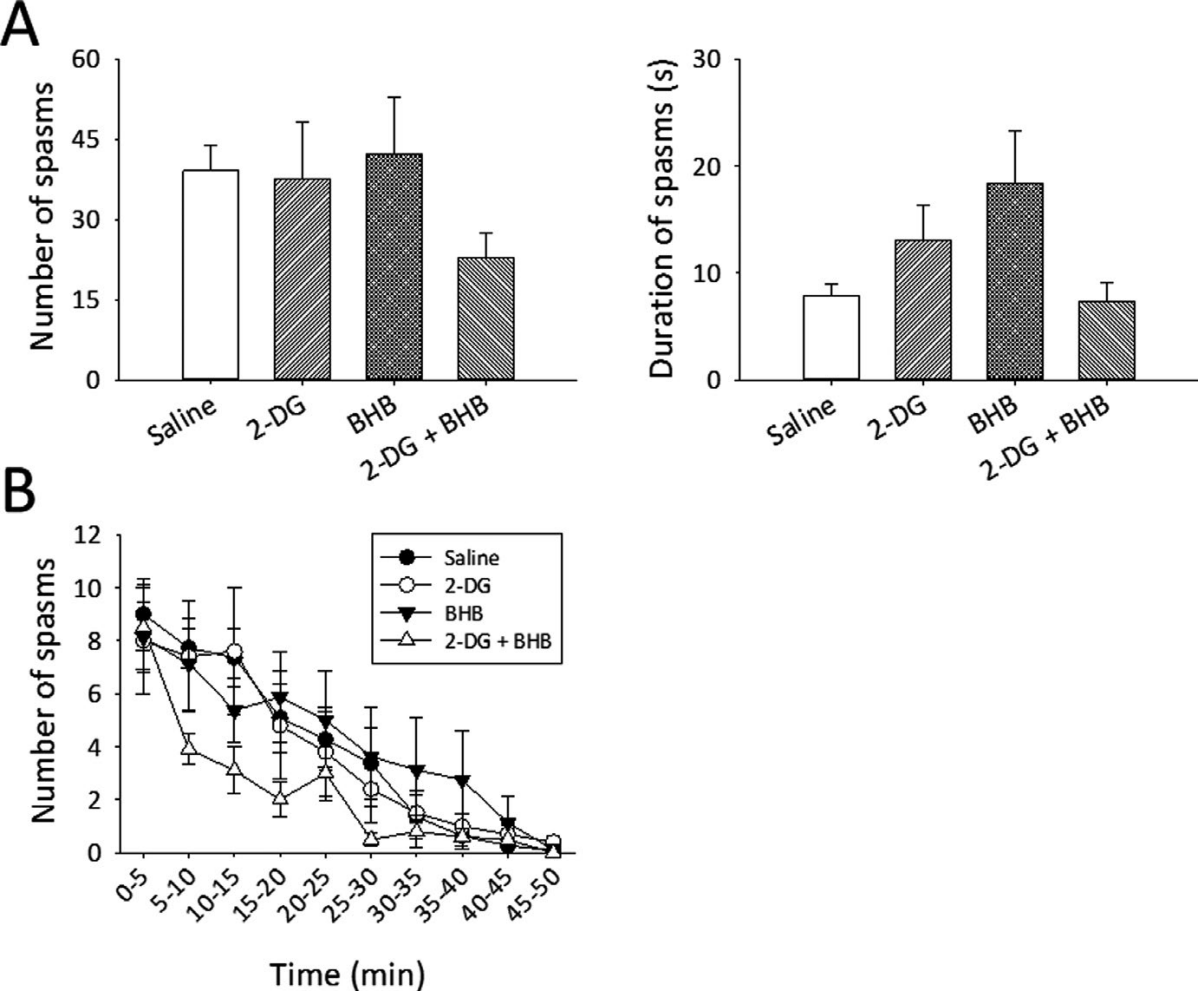
Effects of 2‐DG and BHB on the number, duration, and progression of NMDA‐induced spasms. (A) Mean ± SEM number of spasms for the entire recording period (left) and average duration of individual spasms for each treatment group (right) (saline, n = 11; 2DG, n = 10; BHB, n = 8; 2‐DG + BHB; n = 10). 2‐DG or BHB alone did not reduce the number or duration of the spasms. The combination treatment of 2‐DG and BHB appeared to reduce spasm number and duration, but the reduction was not statistically different from other treatment groups (*P* = .32 and *P* = .12, respectively, one‐way ANOVA). (B) The number of NMDA‐induced spasms gradually decreased with time in a near‐linear manner; 2‐DG or BHB alone did not alter the course. The combination of 2‐DG and BHB seemed to cause a sharper decline in the 10‐20 min epoch. However, this difference is not statistically different from other treatment groups at the same time points (*P* = .56, two‐way repeated‐measures ANOVA, drug treatments × time points)

Video and EEG recordings were analyzed using Sirenia Seizure Pro software (Pinnacle) and checked visually to determine the number and duration of individual spasms. Animals that died in the middle of experiments were excluded from analysis. One‐way analysis of variance (ANOVA) was used to compare the total spasm number and mean duration between the treatment groups (Figure 2A). Two‐way repeated‐measures ANOVA was used to compare the number of spasms measured at different time points (Figure 2B). A statistical difference was defined as *P* < .05.

## 3 RESULTS

### 3.1 Phenotypes of NMDA‐induced behavioral and electrographic spasms

Pups began to show behavioral changes 5‐10 min after NMDA injection—their tails flicked in rapid, sweeping “S” motions, followed by bursts of running, separated by moments of freezing. The first flexion spasm (ie, tail coiled under body, head, and neck hyperflexed, Figure 1C), occurred ~15 min after NMDA injection. In between spasms, rats ran around the cage and sometimes exhibited freezing behaviors. Spasms gradually decreased with time and subsided ~50 min after the first spasm, similar to previous studies.^16^ Electrographically, NMDA caused increased EEG activity with intermixed sharp and slow waves of variable amplitudes, reminiscent of hypsarrhythmia. Spasms often correlated with a sudden decrease and flattening of EEG activity resembling the electrodecrement seen on EEG of IS patients (Figure 1D). In some cases, EEG changes were minimal, though the rat was clearly exhibiting a spasm. Data reported here are based on behavioral spasms.

### 3.2 2‐DG and BHB did not significantly reduce the number or duration of NMDA‐induced spasms

Control (saline‐treated) pups experienced an average of 39.3 ± 4.6 spasms over a period of ~50 min, with spasm duration of 7.8 ± 1.2 s (mean ± SEM, n = 11 rats, Figure 2A). In the 2‐DG‐treated group, the total number spasms and their mean duration were similar to controls (37.6 ± 10.7 and 13 ± 3.3 s, respectively, n = 10 rats). Likewise, the number and duration of spasms were not reduced in BHB‐treated rats (42.2 ± 10.7, 18.4 ± 4.9 s, n = 8 rats). These data suggest that 2‐DG or BHB alone is ineffective in reducing NMDA‐induced spasms. To test whether 2‐DG and BHB together might exert a synergistic antiseizure effect, we administrated both drugs to another group of animals. 2‐DG/BHB‐treated animals exhibited fewer spasms (22.8 ± 4.7, n = 10 rats), but this reduction did not reach statistical significance (*F* = 1.2, DF=3, *P* = .32, one‐way ANOVA, Figure 2A). Spasms duration was also unaltered (*F* = 2.1, DF=3, *P* = .12, one‐way ANOVA, Figure 2A).

### 3.3 2‐DG and BHB did not significantly alter the progression of NMDA‐induced spasms

To characterize the time course of spasms and determine whether 2‐DG or BHB alters the progression or regression of spasms, we binned the number of spasms into 5‐min epochs. The number of spasms gradually declined over time in a near‐linear manner and subsided after ~50 min (Figure 2B). Treatment with 2‐DG or BHB alone did not alter the course of this decrease, as the number of spasms at each time point was similar to saline controls. Treatment using combined 2‐DG and BHB tended to accelerate the decline of spasms (10‐20 min epoch, Figure 2B), but again, this change was not statistically significant compared with other treatment groups (Figure 2B, *F* = 0.94, DF = 27, *P* = .56, two‐way repeated‐measures ANOVA, drug treatments × time points).

## 4 DISCUSSION

This study is the first attempt to use 2‐DG or a combination of two metabolic agents (2‐DG and BHB) to treat spasms in an IS animal model. Our hypothesis that these agents, individually or in combination, would afford spasms reduction was not supported, but the results do provide some useful information for understanding the mechanisms of IS therapy.

First, betamethasone‐NMDA caused consistent, reproducible hyperflexion spasms in P15 rats, correlated with hypsarrhythmia‐ and electrodecrement‐like EEG changes (Figure 1C,D), supporting the use of this model for IS drug screening.

Second, the lack of 2‐DG and BHB effects on spasms occurrence suggests that the underlying mechanisms of betamethasone‐NMDA‐induced spasms are different from other acute seizures induced by chemoconvulsants (eg, pilocarpine) or electrical stimulation (kindling). For example, we previously showed that 2‐DG abrogates pilocarpine‐induced status epilepticus in young animals,^12^ suppresses the progression of kindled seizures,^9^, ^10^ and diminishes epileptiform bursts in hippocampal slices.^10^, ^11^ The mechanisms of 2‐DG's antiseizure action remain elusive and may include inhibition of ATP‐dependent membrane‐bound pumps (eg, Na^+^‐ K^+^ pump, presynaptic H^+^ pump (V‐ATPase]) or increased tonic GABAergic currents.^18^ BHB or other KB has clear antiseizure effects in many seizure models^19^, ^20^ and in clinical studies.^21^ A previous study using the same NMDA IS model showed that multiple BHB injections over 3 days before the NMDA insult (ie, pretreatment) increased the latency and reduced the total number of spasms, while a single injection 30 min before NMDA administration had no effect.^17^ Similarly, our single BHB dose after the first spasm did not have an effect in this model. The mechanisms of KB antiseizure effects are also unclear and may involve ATP‐sensitive potassium channels, presynaptic glutamate loading, or mitochondrial dysfunction.^7^, ^19^, ^22^

The lack of effect of 2‐DG and BHB in the NMDA‐IS model (in contrast to other seizure models) further suggests that IS may not share the same mechanisms with other seizure types that rely on the integrity of energy metabolism and are therefore susceptible to metabolic regulation. It is also possible that an anti‐spasm agent might be model specific; for example, the ovarian steroid, 17 β‐estradiol, suppresses spasms in the *Arx* mutation model^23^ but fails to reduce spasms in the NMDA model^24^ or the multiple‐hit model.^25^ Therefore, the ineffectiveness of 2‐DG and BHB here does not exclude their potential benefit in other models of IS or in patients. In addition, the timing and paradigm of drug administration may also matter. As shown by Yum and colleagues, chronic treatment of BHB for 3 days (but not single dose) after the development of spasms reduces future spasms. Whether chronic treatment with the combination of 2‐DG and BHB can achieve similar anti‐spasm effects remains to be tested.

In conclusion, our study shows that acute metabolic modification of two metabolic pathways, glycolysis and ketogenesis, using 2‐DG, BHB, or their combination does not significantly reduce the severity, frequency, or progression of spasms in the betamethasone‐NMDA model. However, it remains to be tested whether these agents would achieve better efficacy in other preclinical IS models or with other drug administration protocols. Our study supports the use of the betamethasone‐NMDA model for drug screening and highlights the need for ongoing drug development, particularly based on new mechanistic ideas.

## CONFLICT OF INTEREST

None of the authors has any conflict of interests to disclose. We confirm that we have read the journal's position on issues involved in ethical publication and affirm that this report is consistent with those guidelines.
